# An active dressing prevents formation of Staphylococcus aureus biofilm on a mucosal surface

**DOI:** 10.1186/1753-6561-5-S6-P62

**Published:** 2011-06-29

**Authors:** P Parks, M Anderson, M Peterson

**Affiliations:** 1 Exp Clin Pharmacol, 3M/UnivMinn, Minneapolis, USA; 2 ExpClinPharmacol, Univ Minnesota, Minneapolis, USA

## Introduction / objectives

An antiseptic containing dressing is used to minimize the risk of nosocomial infections including Staphylococcus aureus, which can invade via skin or mucosal surfaces. This study developed a mucosal model of S. aureus biofilm formation and determined the effect of the active dressing (Tegaderm**™** CHG) on biofilm formation and mucosal integrity.

## Methods

Explants of normal porcine vaginal mucosa (full-thickness) were infected with biofilm-producing methicillin-resistant S. aureus (MRSA) (3.2x10^5 CFU) for 2h – 48h. Following infection (2h), explants were treated with the active dressing (Tegaderm**™** CHG) for 22-46h or left untreated (controls). Formation of MRSA biofilm was evaluated by scanning electron microscopy. Also, bacteria were enumerated from infected explants, which were washed 3x in PBS by vortex mixing and compared to unwashed explants, to determine the effects of the active dressing (Tegaderm**™** CHG) on MRSA growth.

## Results

MRSA exhibited typical growth on porcine vaginal mucosa. MRSA recovered from infected mucosa at 24h were mainly adherent [washed (adherent): 6.71±0.07; vs. total (planktonic + adherent) 8.27±0.06 log10 CFU/explant]. Biofilm was evident on MRSA-infected vaginal mucosa at 24 h via SEM, and MRSA disrupted the integrity of the mucosal surface. Active dressing (Tegaderm**™** CHG) exposures for 24h reduced the number of MRSA to 3.44±1.00 vs. untreated controls 7.90±0.00 log10 CFU/explant. At 48h, no bacteria were detected in the active dressing (Tegaderm**™** CHG) treated group compared to untreated controls 7.96±0.15 log10 CFU/explant.

## Conclusion

MRSA biofilms can form on normal healthy mucosal tissue. An antiseptic containing active dressing (Tegaderm**™** CHG) prevented MRSA biofilm formation.

## Disclosure of interest

None declared.

